# Predicting the outcome of Thoroughbred stallion matings on the basis of dismount semen sample analyses

**DOI:** 10.1530/REP-22-0309

**Published:** 2023-01-18

**Authors:** Robert John Aitken, Sarah Lambourne, Ashlee Jade Medica

**Affiliations:** 1 Priority Research Centre for Reproductive Science, Discipline of Biological Sciences, School of Environmental and Life Sciences, College of Engineering Science and Environment, University of Newcastle, Callaghan, NSW, Australia; 2 Hunter Medical Research Institute, New Lambton Heights, NSW, Australia

## Abstract

**In brief:**

A capacity to predict the likelihood of pregnancy following natural matings would be of considerable benefit to the Thoroughbred horse breeding industry. In this article, we describe a strategy for achieving this outcome through the analysis of dismount samples, that achieved an overall accuracy of 94.6%.

**Abstract:**

The purpose of this study was to determine whether the analysis of dismount semen samples from Thoroughbred stallions could be used to predict whether a given mating would result in a pregnancy. The analysis was based on 143 matings of 141 mares by a cohort of 7 Thoroughbred stallions over a 4-week period at an Australian Stud. The criteria of semen quality utilized in this analysis involved a preliminary assessment of the raw dismount sample in terms of semen volume, sperm number, and sperm movement characteristics using an iSperm® Equine portable device. Following this initial assessment, a subpopulation of progressively motile spermatozoa was isolated by virtue of the cells ability to migrate across a 5 µm polycarbonate filter in a Samson™ isolation chamber over a 15-minute period. These isolated cells were again evaluated for their number and quality of movement using the iSperm® system and, in addition, assessed for their ability to reduce WST-1, a membrane impermeant tetrazolium salt. These data were then combined with additional information describing the ages of the animals used in this study, their historical breeding records, and mating frequency during the breeding season. The total data set was then used to predict the occurrence of pregnancy, as confirmed by ultrasound at ~14 days post mating. The criteria used to predict fertility in the ensuing multivariate discriminant analysis were optimized for each individual stallion. Using this strategy, we were able to successfully predict the outcome of a cover with an overall accuracy of 94.6%.

## Introduction

As with many species of domestic animal, the selection of Thoroughbred horses on the basis of their athletic prowess has been achieved at a cost to fertility, which is low. Several independent studies of Thoroughbred fecundity have revealed a per cycle conception (PCC) rate of around 60–69% depending on the location of the stud and a variety of modifying factors (Morris & Allen 2002, Lane *et al.* 2016). Overall reproductive performance is known to depend on a number of contributory factors, including the status and age of both the stallion and the mare, the time elapsed between parturition and mating, month of breeding, lactation, genotype, mating frequency, and endocrine status (Madill 2002, Hanlon *et al.* 2012, Lane *et al.* 2016, Ball *et al.* 2019, Todd *et al.* 2020, Castaneda *et al.* 2021).

This relative lack of fertility is clearly a major problem for the Thoroughbred breeding industry. Given the limited duration of the breeding season (from the beginning of September until the end of February or from February to the beginning of July, in the Southern and Northern hemisphere, respectively) and the strong desire for mares to be in foal as early in the breeding season as possible, any covers that do not result in a pregnancy are a major barrier to efficiency. In this context, it would be very helpful to the industry if it could be determined at the time of mating, whether a given cover is likely to result in a pregnancy. If pregnancy is deemed unlikely, then the mare can immediately be mated again during the same cycle, without having to wait 3 weeks for estrus to return. In this study, we have collected ‘dismount’ semen samples from stallions immediately after a cover has occurred and determined whether a detailed analysis of such residual material can result in an accurate prediction of conception and subsequent pregnancy. The results successfully identify sets of criteria describing predominantly sperm number and the quality of sperm movement, which can be used to predict the likelihood that a given cover will result in a pregnancy, with high levels of accuracy. When the diagnostic criteria are optimized for each individual stallion, then predictions of pregnancy were generated that were correct 79–100% of the time. These results provide the Thoroughbred breeding industry with a novel suite of diagnostic tools that will serve to significantly enhance the efficiency of their industry.

## Materials and methods

### Materials

SpermSafe (E)™ (Breed Diagnostics, Newcastle, New South Wales, Australia) was utilized throughout this study for the incubation of spermatozoa. WST-1 was purchased from Sapphire Bioscience (Redfern, NSW, Australia). Equipment and media were maintained at ambient temperatures between 22 and 25°C for the duration of semen collection and processing.

### Study design and sperm preparation

Institutional ethical approval was secured from the University of Newcastle Animal Ethics committee for this project (A-2011-122) which utilized equine dismount samples (*n*  = 143) donated from seven commercial Thoroughbred stallions standing at stud in the Hunter Valley, Australia. Dismount samples were collected during three breeding sessions per day, in early morning (am), lunchtime (noon), and afternoon (pm), over a 1 month period. As the stallion dismounted the mare following breeding, residual semen was gently milked from the urethra and collected directly into a sterile 50 mL specimen jar. The relationship between semen quality in dismount samples and the quality of the entire ejaculate has already been addressed in a previous publication (Gibb *et al.* 2014). This study revealed highly significant correlations between core sperm parameters such as sperm vitality (R^2^ = 0.93), total motility (TM) (R^2^ = 0.92), progressive motility (PM) (R^2^ = 0.85), % rapid cells (R^2^ = 0.80), oxidative stress parameters such as 4HNE (R^2^ = 0.73) and 8OHdG (R^2^ = 0.94), sperm count (R^2^ = 0.54), and tail abnormalities (R^2^ = 0.70), thereby validating the use of such samples in a diagnostic context. The semen was then drawn up into a transfer pipette and carefully expelled into a 15 mL Falcon tube, through a non-woven semen filter pouch (Minitube Australia, Smythesdale, Victoria, Australia) to remove any gel fraction or debris from the sample. Once filtered, the volume was recorded, and the sample was maintained in the dark at ambient temperature.

### Sperm analysis

Sperm motility in both the raw ejaculates and Samson™ isolated samples was objectively determined using iSperm® Equine (Dini *et al.* 2019), a mobile computer-assisted sperm analyzer (mCASA; Aidmics Biotechnology Co., Ltd, Taipei City, Taiwan). The iSperm® equine software and instrumentation included an iPad Mini (Apple Inc., Cupertino, CA, USA) plus the proprietary microscope camera (Aidmics) which were set up according to the guidelines of the iSperm® instruction manual. When testing each sample, a base chip was mounted onto the sample collector before 10 µL of the sample was collected using a micropipette and placed onto the surface of the base chip. The sample collector was then flipped over into the cover chip and pressed on the countertop to click together, as per the instruction manual. The base chip plus cover chip were then screwed into the microscope, with a heat block attached, and connected to the iPad Mini camera to be analyzed. Each sperm sample (initial dismount sample or post-Samson™ sample) was analyzed a minimum of three times consecutively in the iSperm® application. The parameters quantified and collected in the iSperm® application included TM (%), PM (%), curvilinear velocity (VCL; µm/s), average path velocity (VAP; µm/s), straight line velocity (VSL; µm/s), straightness (STR; VSL/VAP), linearity (LIN; VSL/VCL), and concentration (million/mL). The following settings were used: 15 frames acquired at 30 frames/s, progressive VAP threshold of 50 µm/s, slow (static) cell VAP threshold of 20 µm/s, slow (static) cell velocity threshold of 0 µm/s, and threshold STR of 75%. Cells exhibiting a VAP of >50 µm/s and an STR of >75% were considered progressive. The isperm device has been validated for use with stallion spermatozoa and the thresholds for PM cited earlier are those recommended in the literature (Dini *et al.* 2019).

### Sperm isolation in the Samson™ System and WST-1 reduction

**Figure 1:**
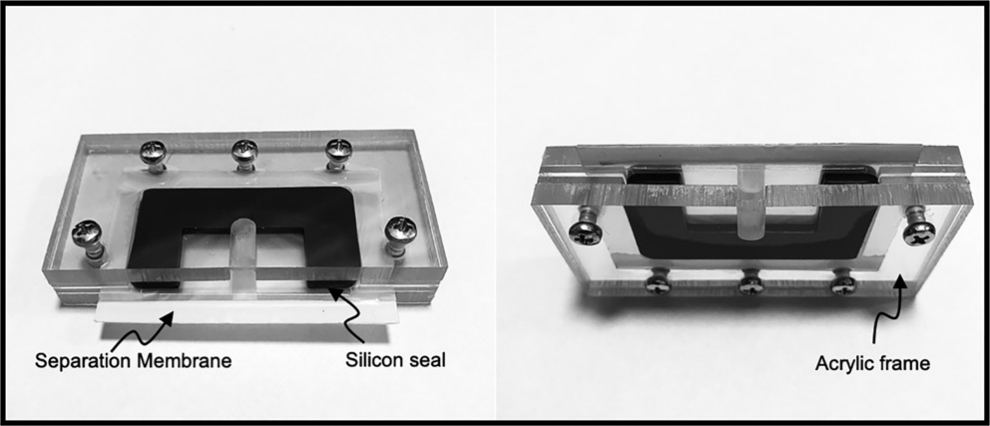
The Samson separation chamber. The device consists of two 650 µL chambers separated by a semipermeable polycarbonate membrane containing pores 5 µm in diameter. Encased in an acrylic frame, the overall dimensions of the device are 75 mm × 45 mm × 25 mm.

The Samson™ System (Memphasys, Sydney, NSW, Australia) consists of a sample chamber and a reaction chamber, separated by a 5 µm polycarbonate filter (Fig. 1). A 0.5 mL aliquot of SpermSafe (E)™ medium (Breed Diagnostics) containing WST-1 (Sapphire Bioscience) at 0.5 mg/mL was deposited into the reaction chamber, immediately followed by deposition of a 0.5 mL aliquot of the filtered dismount sample into the sample chamber. WST-1 is a membrane-impermeant probe, which in the presence of equine spermatozoa becomes reduced to a formazan product that can be readily detected spectrophotometrically. WST-1 activation does not appear to require an intermediate electron acceptor in the presence of equine spermatozoa and, instead, involves the direct reduction of WST-1 by electrons released at the cell surface (Berridge & Tan 2000, Berridge *et al.* 2005). We, therefore, reasoned that WST-1 reduction would not only be an efficient means of determining sperm number but might also reveal something of their underlying biochemical status. Following the addition of a dismount sample, the Samson™ system was kept at ambient temperature for 15 min during which time spermatozoa were able to swim across the separation membrane and into the reaction chamber. Following this, 250 µL of the sample was carefully removed from the reaction chamber and placed into a clean 1.5 mL Eppendorf tube. One hundred microliter aliquots were then transferred in duplicate into a 96-well plate and absorbance was read immediately at 450 nm on a Spectrostar nano (BMG Labtech, Mornington, Victoria, Australia) with SpermSafe (E)™ plus WST-1 used as a media blank control. While the absorbance was being read, the remaining 50 µL of the isolated sample was used to analyze motility and cell concentration as described earlier using the iSperm® Equine system.

### Animal information collection

At the end of the breeding season, the following data were collected: stallion and mare ages, mare fecundity, and historical breeding efficiency (the ratio of 30-day pregnancies over the mare’s breeding years based on data from the Australian Stud Book website; for example, if a mare went to stud each year for 5 years and carried 3 pregnancies to a minimum of 30 days, she would receive a score of 0.6), as well as weekly PCC rates of stallions, mating frequency, and cumulative number of covers for the duration of the study and the season. Cross-covers, where the mare was covered more than once by the same stallion in the same estrous cycle, were excluded from the discriminant analysis. All dismount samples were recovered from the last mating before ovulation.

### Statistical analysis

All data were analyzed using JMP Pro16 software (SAS Institute Inc 2004, Cary, NC, USA) using a variety of methods including contingency table analysis followed by both Pearson and Likelihood ratio chi-square tests, ANOVA, linear regression, stepwise discriminant analysis, and receiver operating curve (ROC) determinations where appropriate. The area under the curve (AUC) was used as a measure of the predictive power of a variable provided by the ROC method. AUC values range from 0.5 (when the explanatory variable is not associated with the analyzed outcome) to 1 (when the variable with the chosen threshold value can distinguish two possible outcomes in 100% of cases). Similarly, linear discriminant function analysis was then used to determine whether any of the parameters of semen quality measured in these dismount semen samples could accurately predict whether a given mating was going to result in a pregnancy. With this form of analysis, 50% accuracy would suggest random selection, while 100% accuracy would indicate that the selected variables could perfectly discriminate fertile from infertile matings. For this purpose, we limited the number of variables that could be drawn into the discriminant function analysis to a maximum of eight. Data are presented as mean ± s.e.m. and for all analyses, *P* < 0.05 was considered statistically significant.

## Results

### Fertility

This analysis was based on 143 covers involving a cohort of seven stallions. Of these 143 covers, 103 (72%) resulted in a positive early pregnancy test at 14–16 days post cover and only 3 of these pregnancies did not remain positive into mid-pregnancy, 30 days post cover (70% positive pregnancy rate).

### Age

The stallions ranged in age from 4 to 18 years (mean 9.3 ± 1.8 years; *n*  =7), and this criterion did not correlate with the attainment of pregnancy. A total of 141 different mares, exhibiting an average age of 9.2 ± 0.3 years, were inseminated in this program (2 mares were re-inseminated after an initial negative cover). Again, there was no significant difference between the ages of the mares that did (9.2 ± 0.4 years: *n*  = 103) and those that did not (9.3 ± 0.6 years; *n* = 38) return a positive pregnancy test.

### Mare status

Of the 143 matings, 35 were to mares that were ‘dry’ (no existing foal), 86 were ‘wet’ (mare being bred with a current foal at foot), and 22 were maiden (never previously given birth to a foal). The status of the mare did not correlate with the incidence of pregnancy over the study period (*P*  > 0.05, chi-square test). The incidence of pregnancy was also not significantly associated with the historical breeding record of the mare. The uniform quality of the mares was indicated by the fact that only 4 of the 143 matings were repeat matings to mares that had failed to conceive following a previous cover.

### Stallion performance

**Figure 2:**
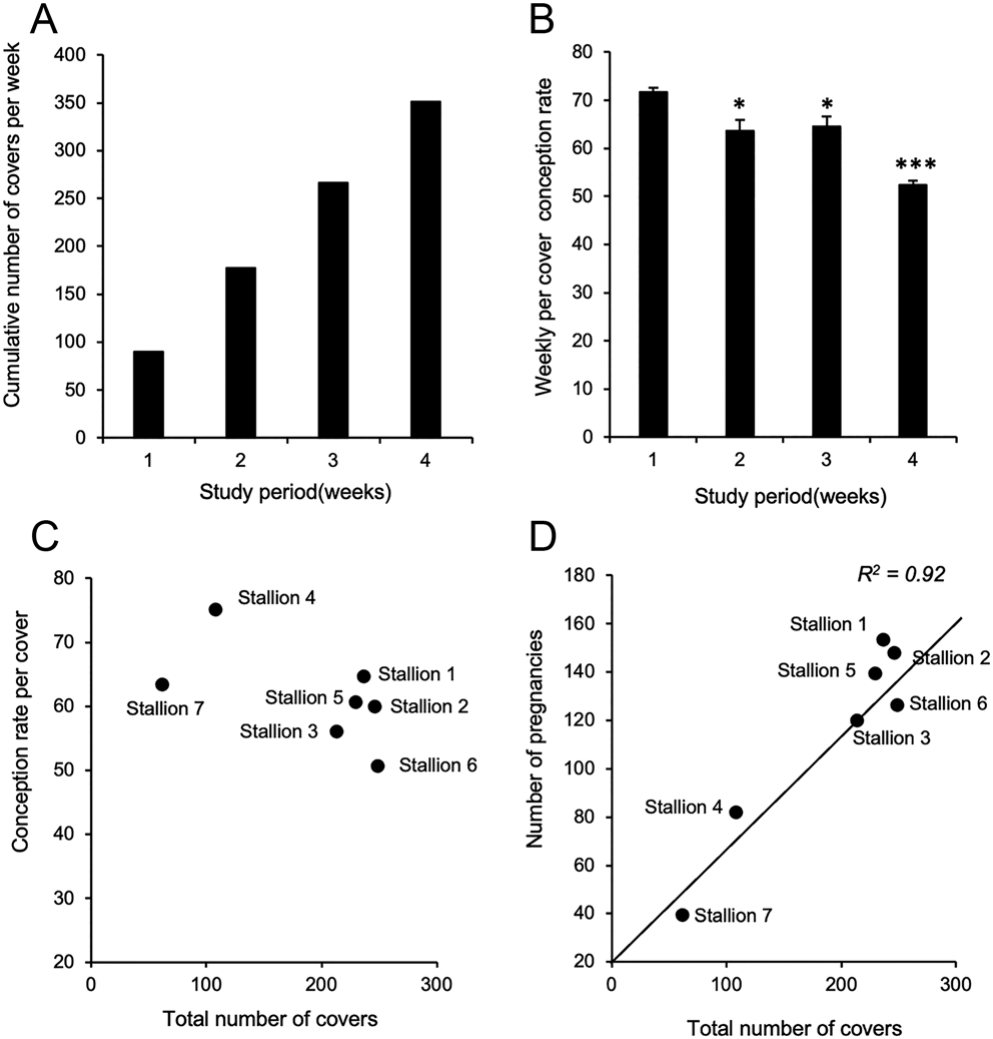
Performance of the Thoroughbred stallions used in this study. (A) Over the 4-week study period, the cumulative number of matings increased at a rate of 87.7 ± 1.1 covers a week. (B) The weekly per cover conception rate significantly declined over the study period in association with the increased number of inseminations. (C) Over the entire season, significant differences in stallion fertility (conception rate per cover) were evident that were not statistically related to the number of covers performed (ejaculation frequency). (D) Across the entire season, the number of covers and the number of pregnancies resulting from those covers increased in a linear fashion, deviations from the regression line reflecting differences in the relative fertility of each stallion. *P < 0.05; ***P < 0.001.

The pregnancy rates observed during the study period varied between stallions – but not significantly (*P*  > 0.05, chi-square test). However, the per stallion conception rate observed during the study period did correlate significantly with the rate for each stallion over the entire season (Table 1) (*r* ^2^ = 0.73; *P* < 0.05) by the end of which, significant differences between stallions were evident (*P*  < 0.001, chi-square test) with Stallion 4 being particularly fertile and Stallion 6, expressing significantly lower levels of fertility than the other stallions (*P*  < 0.05; Analysis of Means for Proportions) (Table 1). It is also evident from this table that the fertility rates recorded over the entire season were lower than those observed during the study period. In this context, ejaculation frequency may be a factor because even with the 4-week study period, we saw a significant time-dependent decline in per cover conception rate for this cohort of stallions as the cumulative number of matings increased at a rate of 87.5 ± 1.1 covers per week (Fig. 2A and B). While this suggests ejaculation frequency may have some modifying impact on fertility rate, this parameter alone could not explain the significant differences in per cover conception rate that were evident between stallions by the end of the season (Fig. 2C). In general, the more covers in the breeding season, the higher the number of conceptions (R^2^ = 0.92; Fig. 2D); the variation around this regression line reflecting differences between stallions in their fundamental fertility.

**Table 1:** Pregnancy rates achieved by individual stallions.

Stallion no.	Total covers	Number pregnant^†^	Number non-pregnant	Percentage success rate (%)	Fertility rate per serve for entire season
1	24	19	5	79	65
2	36	26	10	72	60
3	21	13	8	62	56
4	18	16	2	89	75*
5	28	18	10	64	61
6	6	4	2	67	51*
7	10	7	3	70	63

*P < 0.05; ^†^At 14 –16 days post cover.

### Dismount semen quality

Dismount samples were successfully collected from all of the matings monitored during the study period, generating a mean volume of 3.2 ± 0.2 mL, containing 52.6 ± 4.7 × 10^6^ spermatozoa/mL that were 30.1 ± 1.7% motile.

**Figure 3:**
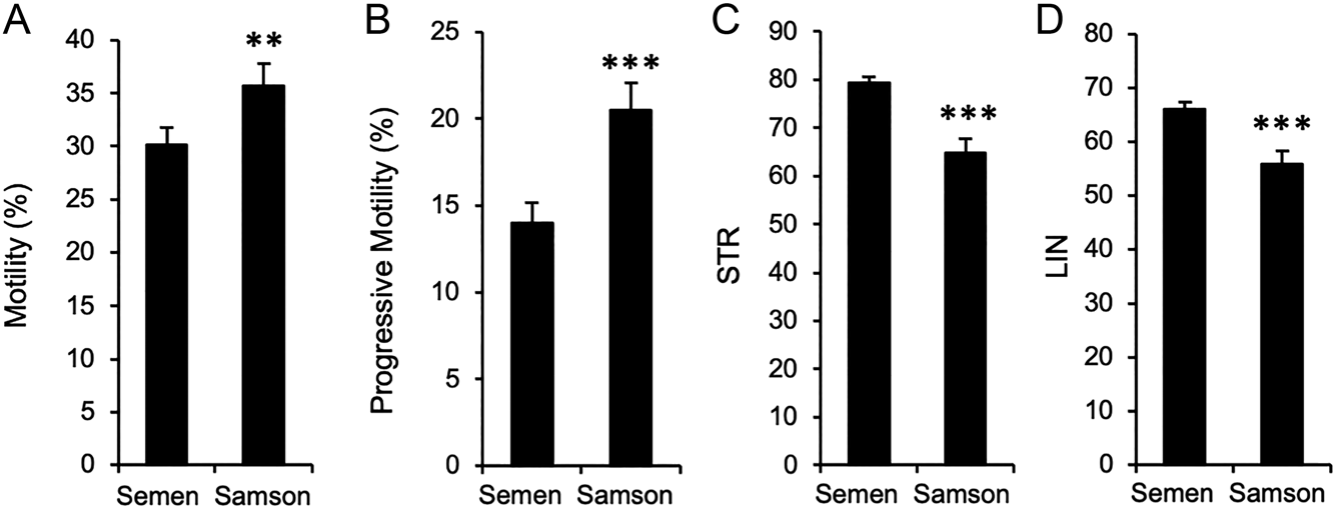
Selection of spermatozoa in a Samson™ chamber. (A) The spermatozoa selected by a 15 min incubation in a Samson™ chamber possessed significantly more motility than the parent dismount semen sample. (B) Progressive motility was also elevated in these Samson™ – isolated sperm populations. (C) However, the STR values were significantly reduced following Samson™ isolation, as were (D) the LIN values. **P < 0.01; ***P < 0.001.

In an attempt to mimic the selection of spermatozoa as they ascend the female reproductive tract, the ability of the spermatozoa from these dismount samples to pass through a polycarbonate separation membrane containing 5 µm pores was assessed in disposable chambers (Samson™ chambers, Memphasys) as described in ‘Materials and methods’. Using an incubation time of 15 min, a mean of 3.6 ± 0.5 × 10^6^ spermatozoa/mL were isolated that were significantly more motile than the parent population (Fig. 3A) and exhibited higher levels of PM (Fig. 3B). These cells were also less linear in their motion than the spermatozoa initially recovered in the dismount sample exhibiting significantly (*P*  < 0.001) reduced levels of STR and LIN (Fig. 3C and D) although the underlying fundamental velocity parameters (VCL, VSL, and VAP) were not significantly changed.

**Figure 4:**
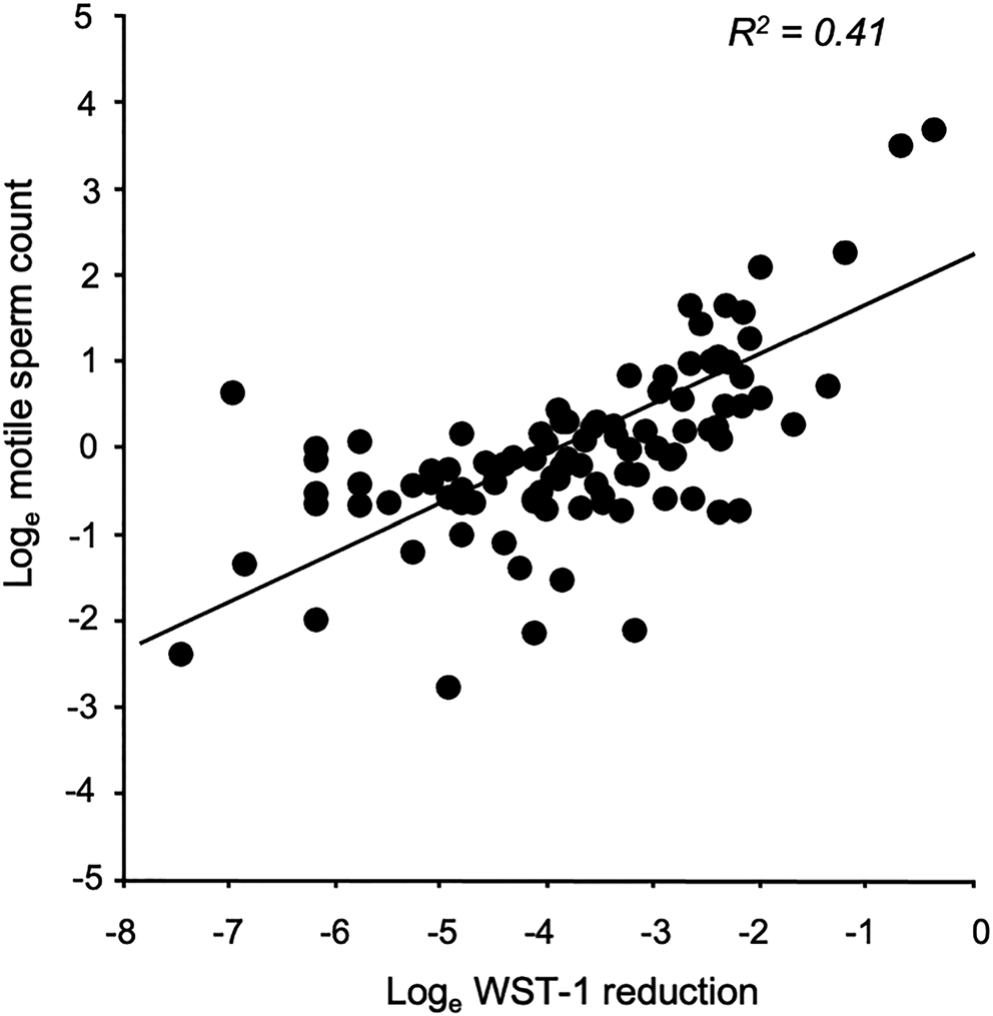
WST-1 reduction by spermatozoa isolated in a Samson™ chamber was significantly correlated with the total number of motile cells (***P < 0.001).

In addition to this sperm migration assay, we also examined the ability of these isolated spermatozoa to reduce the tetrazolium salt, WST-1, as another marker of equine sperm function. As anticipated, WST-1 reduction by these isolated sperm populations was found to generate a significant correlation (*P*  < 0.001; *R* ^2^ = 0.41) with motile sperm count (Fig. 4).

### Prediction of pregnancy

If the analysis was confined to the age of the stallion, sperm number, and movement characteristics in the dismount semen sample, then stepwise analysis drew the following parameters into the discriminant function equation (stallion age, volume of the dismount sample, sperm concentration, TM, PM, VCL, STR, and LIN) but only achieved an overall accuracy of 65.5% (34.5% of samples were misclassified) and an AUC value of 0.72 in the ROC analysis. If this analysis was supplemented with the WST-1 reduction data on Samson™ isolated cells, then neither the accuracy of the prediction (68.3%) nor the ROC, AUC value (0.71) was significantly changed.

**Figure 5:**
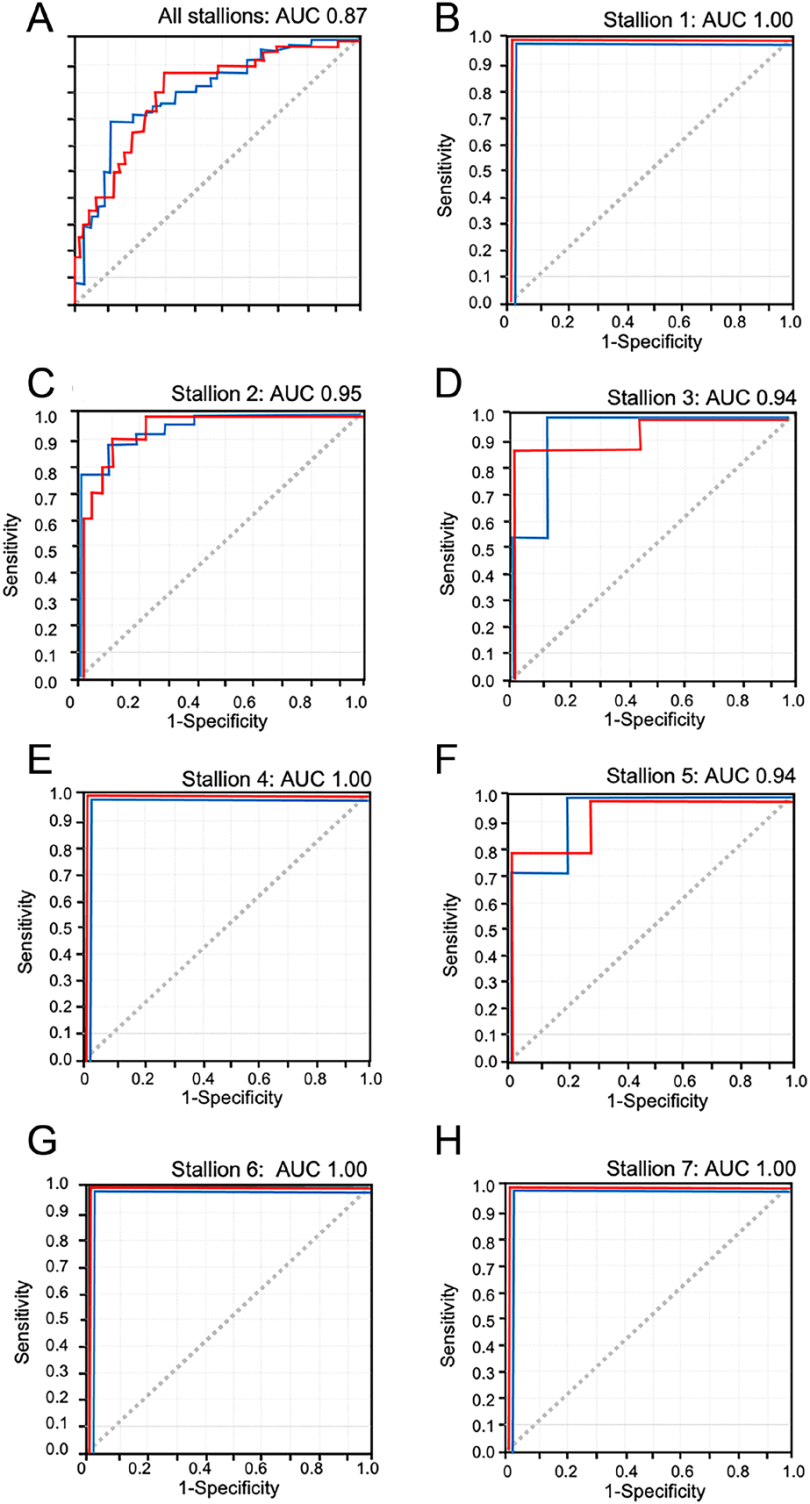
Receiver operating curves for the prediction of pregnancy based upon the analysis of dismount samples. For such analyses, the area under the curve (AUC) values should be as close to 1.0 as possible. (A) Prediction of fertility across the entire data set generated a high AUC value of 0.82 that was only limited by the variation in fundamental fertility between stallions. If this variable was taken out of the equation by optimizing the analysis for each individual stallion (B–H), then AUC values in excess of 0.94 were routinely obtained, indicating an extremely high level of predictive accuracy.

If all continuous variables (including the movement characteristics of the spermatozoa and their capacity to reduce WST-1 following isolation in the Samson™ system) were provided to the discriminant analysis and a step-wise analysis was performed, then the first eight variables incorporated into the regression equation were dismount semen volume, VCL in semen, LIN in semen, WST-1 absorbance, post-Samson™ PM, post-Samson™ VAP, total number of covers for the season, and Samson™ sperm concentration (full discriminant function equation given in the Supplementary file, see section on supplementary materials given at the end of this article). This equation incorrectly classified the outcome of 37/143 matings, giving a successful prediction rate of 73.76%, while the ROC analysis revealed an AUC of 0.87 for both the positive and negative prediction of fertility (Fig. 5A).

Since there were significant differences in reproductive success between stallions over the breeding season, the discriminant analysis was repeated for each individual stallion. The results of this analysis for each stallion are presented in Tables 2 and 3. Table 2 describes some key elements of the dismount semen quality for each stallion while Table 3 presents the criteria that were used by the stepwise linear discriminant analysis to predict whether a given insemination would be successful in establishing a pregnancy, the overall success of the discriminating process and the AUC values for the ROC analyses. A slightly different set of criteria were used to optimize the predictive process for each stallion, but, in general, they described the quality of the dismount sample in terms of volume, sperm count, movement characteristics, and the quality of the migrated sperm population recovered from the Samson™ cartridges. In one case (Stallion 4), the age of the mare was also drawn into the discriminant analysis and interestingly the mean age of the mares served by this stallion (11.1 ± 0.9) was significantly higher and more variable than all of the other stallions put together (8.9 ± 0.3; *P* < 0.05). Predicting the fertility of Stallions 5 and 7 also brought WST-1 reduction into the regression equation, but this was not the case with the other stallions; much (but not all) of the information contained within this criterion, presumably being taken up by the parallel measurements of sperm count and movement characteristics. Using these simple criteria, we could predict the outcome of any given cover with a high degree of accuracy (Table 2) with an AUC in the ROC analysis approximating to 1 (Fig. 5B, C, D, E, F, G and H). For Stallions 1–7, analysis of the dismount sample provided enough information to predict the outcome of a given mating with 100, 88.6, 95.2, 100, 78.6, 100, and 100% accuracy, respectively, an average of 94.6% for each stallion. Overall, when the analysis was optimized for each individual stallion in this way, we were able to predict the outcome of a given cover with 91.6% accuracy (131/143 covers).

**Table 2:** Quality of the dismount samples for individual stallions.

Stallion no.	Volume (mL)	Count (10^6^)	Total motility (%)	Prog motility (%)	VCL (µm/s)	VAP (µm/s)	VSL (µm/s)	STR (%)	LIN (%)
1	3.9 ± 0.5	23.1 ± 2.7	20.2 ± 2.4	8.3 ± 1.1	70.6 ± 3.7	56.9 ± 3.8	48.8 ± 3.8	80.7 ± 2.0	66.2 ± 2.6
2	2.1 ± 0.3	40.8 ± 4.0	31.3 ± 2.9	14.6 ± 1.9	62.9 ± 2.5	54.6 ± 2.5	48.1 ± 2.4	83.9 ± 2.6	73.6 ± 2.6
3	5.4 ± 0.9	33.7 ± 6.5	32.7 ± 4.7	12.8 ± 2.4	73.1 ± 4.3	56.4 ± 3.9	46.8 ± 3.6	75.6 ± 4.0	59.2 ± 3.5
4	3.6 ± 0.4	52.0 ± 10.3	26.6 ± 4.3	11.9 ± 2.7	66.9 ± 4.4	54.7 ± 4.1	46.7 ± 3.7	79.8 ± 3.9	65.6 ± 3.6
5	3.1 ± 0.3	86.8 ± 7.1	37.4 ± 4.1	20.2 ± 3.4	71.9 ± 3.8	59.9 ± 3.9	50.8 ± 3.7	78.5 ± 3.2	65.6 ± 3.2
6	1.6 ± 0.5	34.5 ± 8.1	24.3 ± 7.9	6.7 ± 2.9	60.2 ± 8.4	42.4 ± 6.3	32.0 ± 4.9	63.2 ± 7.5	46.1 ± 6.0
7	1.5 ± 0.3	121.2 ± 49.0	33.1 ± 9.5	19.0 ± 7.9	84.8 ± 5.1	70.4 ± 6.2	57.3 ± 6.3	78.7 ± 1.9	65.4 ± 3.3

**Table 3:** Criteria employed to predict the success of insemination by stallion.

Stallion ID/criteria employed	Accuracy of prediction (*n*)	ROC analysis AUC
1	100% (24)	1.00
Volume of dismount sample		
Sperm conc in dismount sample		
VSL in dismount sample		
STR in dismount sample		
Total motility in dismount sample		
Sperm conc following migration*		
Total motility following migration		
Progressive motility following migration		
2	88.6% (36)	0.95
Sperm conc in dismount sample		
Total motility in dismount sample		
Progressive motility in dismount sample		
STR in dismount sample		
LIN in dismount sample		
Total motile count in dismount sample		
STR following migration		
LIN following migration		
3	95.2% (21)	0.94
Volume of dismount sample		
Sperm conc in dismount sample		
Total motility in dismount sample		
Progressive motility in dismount sample		
VAP in dismount sample		
Progressive motility following migration		
VCL following migration		
Total motile count following migration		
4	100 (18)	1.00
Volume of dismount sample		
Progressive motility in dismount sample		
VCL in dismount sample		
LIN in dismount sample		
Sperm count following migration		
VAP following migration		
STR following migration		
Mare age		
5	78.6% (28)	0.94
Volume of dismount sample		
Sperm conc in dismount sample		
LIN in dismount sample		
WST-1 reduction following migration		
Progressive motility following migration		
STR following migration		
LIN following migration		
Total motile sperm count following migration		
6	100% (6)	1.00
Total motility in dismount sample		
Total motile count in dismount sample		
STR following migration		
Sperm conc following migration		
7	100% (10)	1.00
Volume dismount sample		
Sperm conc in dismount sample		
Progressive motility in dismount sample		
STR in dismount sample		
LIN in dismount sample		
Progressive motility following migration		
Sperm conc following migration		
WST-1 reduction following migration		

*Migration in the Samson™ chamber.

## Discussion

The results of this analysis demonstrate that the quality of Thoroughbred stallion semen has a major impact on the likelihood that a natural cover will result in a pregnancy. Moreover, the quality of the entire ejaculate appears to be strongly correlated with the attributes of a dismount sample collected on withdrawal. These observations open up the possibility that rapid analysis of such samples will enable Thoroughbred breeders to determine whether a cover is likely to result in a pregnancy and thus whether the mare needs to be cross covered during the same estrous cycle to ensure a positive outcome. While female factors such as age and breeding history are absolutely acknowledged to be important determinants of pregnancy outcome in the Thoroughbred breeding industry (Gibb *et al.* 2014), the results obtained in this study suggest that while the age of the mare may occasionally have an impact, the major determinant of a successful mating is semen quality.

Importantly the high level of predictive accuracy achieved in this study could not have been achieved by a conventional semen analysis focusing on the concentration of spermatozoa and their movement characteristics. It is possible that a detailed analysis of sperm morphology might have increased the predictive accuracy of such semen profiling (Gravance *et al.* 1997). However, this would have involved time-consuming laboratory assessments and our intention is to develop rapid assessment strategies that can be used in the field. Using semen quality alone, we secured a predictive accuracy of 65.5%, which is certainly better than the no discrimination 50% value, but not high enough to be of practical value.

In order to achieve diagnostic significance, it was necessary to supplement such conventional assessments of semen quality with detailed analyses of the spermatozoa isolated using a Samson™ chamber. This simple system assesses the ability of equine spermatozoa to cross a separation membrane containing 5 µm pores over a 15-min period. The isolated populations were more motile than the parent population with higher levels of PM but lower values for STR and LIN. This modified pattern of movement suggests a reduction in the LIN and STR of sperm progression consistent with the initiation of a more hyperactivated form of motility in this subpopulation of isolated cells (McPartlin *et al.* 2009). These isolated cells were also shown to be capable of directly reducing the membrane impermeant tetrazolium salt, WST-1, without the need for a low potential intermediate electron acceptor, as is generally the case (Berridge & Tan 2000). This reductive event appears to be taking place directly at the cell surface, presumably as a result of electrons released by the oxidation of NADH via a plasma membrane electron transport system (Berridge & Tan 2000). The purpose of this enzyme system is thought to involve the maintenance of intracellular NADH/NAD^+^ redox balance and the consequential regulation of glycolytic flux (Gray *et al.* 2011). Since WST-1 reduction is highly dependent on cell viability and number, we reasoned that it might be a very convenient means of assessing motile sperm concentration as indeed, did turn out to be the case (Fig. 4).

When sperm count and motility in the raw dismount sample were combined with data describing the WST-1 reducing capacity of spermatozoa following isolation in the Samson™ device, the ability to predict the fertility outcome was only slightly increased from 65.5 to 68.3%. However, if we incorporated the movement characteristics of the cells following Samson™ isolation and data on the total number of covers for the season, then the accuracy of the prediction was increased to 73.76%. Ejaculation frequency certainly had an impact on the conception rates observed for this cohort of stallions. Even within the study period, the weekly PCC rate declined as the number of covers increased (Fig. 2A and B). However, there were also significant differences between stallions in their relative fertility that was not statistically correlated with the cumulative number of covers undertaken by each stallion in the course of the breeding season. Because of this difference between stallions in relative fertility, we undertook to optimize the specific parameters of semen quality used to predict fertility on an individual stallion basis. This approach dramatically changed the predictive value of these dismount sample analyses, generating an overall successful classification rate of 94.6% for each stallion and 91.6% per cover. The criteria selected to achieve this high level of predictive accuracy are presented in Table 2, and the discriminant function equations are revealed in the Supplementary material accompanying this manuscript. ROC analyses confirmed the high predictive value of the selected variables, generating AUC values in excess of 0.94 for all stallions in the study group.

In general, these predictive parameters involved elements of the traditional semen profile (volume, TM, and count), the movement characteristics of spermatozoa in semen and their movement following isolation in the Samson™ chamber. In one instance (Stallion 4), female age was brought into the equation to reflect the age composition of the mares covered by this particular male. In two other stallions (5 and 7), WST-1 reduction was drawn into the discriminant function equation, presumably reflecting the ability of this probe to reflect some key elements of sperm metabolism. In total, this analysis reveals that the factors limiting stallion fertility vary slightly between individuals, making the construction of generalized fertility prediction algorithms difficult. However, if the analysis is optimized on an individual stallion basis, then discriminant function equations can be written that reflect the fertilizing potential of an ejaculate and predict the chances of establishing a pregnancy with great accuracy. This facility should be of considerable value to the Thoroughbred breeding industry in managing the reproductive performance of its valuable livestock.

## Supplementary Material

Supplementary Material

## Declaration of interest

RJA, AM and SL are supported by Memphasys Ltd. R J Aitken is on the editorial board of Reproduction. R J Aitken was not involved in the review or editorial process for this paper, on which he is listed as an author.

## Funding

This work was funded by Memphasys Ltd.

## Author contribution statement

SL undertook a majority of the work described in this study and AM developed and validated the WST-1 assay. RJA designed the study and prepared the initial manuscript draft. All authors provided intellectual input and critically edited the article.
