# Supplementary material for: Predicting the outcome of Thoroughbred stallion matings on the basis of dismount semen sample analyses
Source: Reproduction. 2023 Jan 18;165(3):281–8. doi: 10.1530/REP-22-0309 (PMC9874971; doi:10.1530/REP-22-0309)
Supplement: Supplementary Material [file supplementary_material.pdf]

Supplementary file - Discriminant function equations

1. **Equation generated when all continuous variables were made available on all stallions achieving a predictive accuracy for the whole group of 73.76%.**

$$\begin{pmatrix} 0.1965089779 \bullet \text{Dismount Semen volume} \\ + 0.0226703655 \bullet \text{Initial VCL} \\ + 0.0086876366 \bullet \text{Total covers for season} \\ + 7.1811893049 \bullet \text{WST-1 reduction} \\ + -0.042306262 \bullet \text{Initial LIN} \\ + 0.0324162773 \bullet \text{Post PM} \\ + 0.0063984396 \bullet \text{Post VAP} \\ + -0.065561492 \bullet \text{iSperm isolated} \end{pmatrix} - 2.3086117306$$

2. **Equation optimized for Stallion 1: predictive accuracy of 100%**

$$\begin{pmatrix} -0.162886299 \bullet \text{Dismount Semen volume} \\ + 0.1490464447 \bullet \text{Initial TM} \\ + 0.0416130554 \bullet \text{Initial VSL} \\ + -0.130209298 \bullet \text{Initial STR} \\ + 0.0189280403 \bullet \text{Post TM} \\ + 0.0228204184 \bullet \text{Post PM} \\ + -0.087584865 \bullet \text{iSperm initial} \\ + 0.2177938904 \bullet \text{iSperm isolated} \end{pmatrix} - (-6.97088411)$$

3. Equation optimized for Stallion 2: predictive accuracy of 88.6%

$$\left(\begin{array}{l} 0.0655604033 \bullet \text{iSperm initial} \\ + -0.061522018 \bullet \text{Initial TM} \\ + 0.0275543575 \bullet \text{Initial PM} \\ + 0.0851884146 \bullet \text{Initial STR} \\ + -0.073316696 \bullet \text{Initial LIN} \\ + -0.080998882 \bullet \text{Post STR} \\ + 0.0712550505 \bullet \text{Post LIN} \\ + 0.3152285504 \bullet \text{Post TMC isperm} \end{array}\right) - 2.0377218639$$

4. Equation optimized for Stallion 3: predictive accuracy of 95.2%

$$\left(\begin{array}{l} -0.245764616 \bullet \text{Dismount Semen volume} \\ + 0.0139995328 \bullet \text{Initial TM} \\ + -0.167930472 \bullet \text{Initial PM} \\ + 0.0253060352 \bullet \text{Initial VAP} \\ + -0.070602976 \bullet \text{Post PM} \\ + 0.031519078 \bullet \text{Post VCL} \\ + 0.4686639992 \bullet \text{Post TMC isperm} \\ + -0.004494141 \bullet \text{iSperm initial} \end{array}\right) - (-0.409980054)$$

5. Equation optimized for Stallion 4: predictive accuracy of 100%

$$\left(\begin{array}{l} -1.029951093 \bullet \text{Dismount Semen volume} \\ + -0.385047226 \bullet \text{iSperm isolated} \\ + 0.2029740852 \bullet \text{Initial PM} \\ + 0.0340640489 \bullet \text{Initial VCL} \\ + -0.144175986 \bullet \text{Initial LIN} \\ + -0.052446446 \bullet \text{Post VAP} \\ + 0.0386563322 \bullet \text{Post STR} \\ + 0.6475871886 \bullet \text{Mare Age} \end{array}\right) - (-3.338335422)$$

6. Equation optimized for Stallion 5: predictive accuracy of 78.6%

$$\left(\begin{array}{l} -0.514553234 \bullet \text{Dismount Semen volume} \\ + -0.028408702 \bullet \text{iSperm initial} \\ + -0.07309869 \bullet \text{Initial LIN} \\ + 0.0144847321 \bullet \text{Post PM} \\ + 5.8260487256 \bullet \text{WST-1 reduction} \\ + 0.0924540322 \bullet \text{Post. STR} \\ + -0.067722137 \bullet \text{Post LIN} \\ + 0.9612232741 \bullet \text{Post TMC isperm} \end{array}\right) - (-4.373918505)$$

7. Equation optimized for Stallion 6: predictive accuracy of 100%

$$\begin{pmatrix} 0.8448584965 \bullet \text{Initial TM} \\ + 0.0784496176 \bullet \text{Post STR} \\ + -47.50159653 \bullet \text{iSp TMC} \\ + 8.6596714532 \bullet \text{iSp Post Conc} \end{pmatrix} - (-0.942551738)$$

8. Equation optimized for Stallion 7: predictive accuracy of 100%

$$\begin{pmatrix} 114.06904232 \bullet \text{Dismount semen volume} \\ + 0.998384278 \bullet \text{iSp Initial} \\ + -11.22583781 \bullet \text{iSp Post} \\ + 378.62880117 \bullet \text{WST-1 reduction} \\ + -6.099484106 \bullet \text{Initial PM} \\ + 20.766470787 \bullet \text{Initial STR} \\ + -1.953276169 \bullet \text{Initial LIN} \\ + 3.5336376238 \bullet \text{Post PM} \end{pmatrix} - 1719.5546059$$

Key

Initial = original dismount sample
Post = Following sperm isolation in a Samson™ chamber
iSperm® Post = sperm concentration and motility following migration in Samson™ chamber
iSperm® Initial = sperm concentration and motility in original dismount semen sample
TM = Total motility
PM =Progressive motility
TMC = Total Motile Count
